# Citrus Flavanone Effects on the Nrf2-Keap1/GSK3/NF-κB/NLRP3 Regulation and Corticotroph-Stress Hormone Loop in the Old Pituitary

**DOI:** 10.3390/ijms25168918

**Published:** 2024-08-16

**Authors:** Marko Miler, Jasmina Živanović, Sanja Kovačević, Nevena Vidović, Ana Djordjevic, Branko Filipović, Vladimir Ajdžanović

**Affiliations:** 1 Department of Cytology, Institute for Biological Research “Siniša Stanković”—National Institute of the Republic of Serbia, University of Belgrade, 11060 Belgrade, Serbia; jasminap@ibiss.bg.ac.rs (J.Ž.); brankof@ibiss.bg.ac.rs (B.F.); avlada@ibiss.bg.ac.rs (V.A.); 2 Department of Biochemistry, Institute for Biological Research “Siniša Stanković”—National Institute of the Republic of Serbia, University of Belgrade, 11060 Belgrade, Serbia; sanja.kovacevic@ibiss.bg.ac.rs (S.K.); djordjevica@ibiss.bg.ac.rs (A.D.); 3 Centre of Research Excellence in Nutrition and Metabolism, Institute for Medical Research, National Institute of the Republic of Serbia, University of Belgrade, 11000 Belgrade, Serbia; nevena.vidovic@imi.bg.ac.rs

**Keywords:** hesperetin, naringenin, ACTH, SOD, CAT, GPx, GR, Interleukin 1 and 6, TNF-α, NLRP3

## Abstract

Oxidative stress and inflammation are significant causes of aging. At the same time, citrus flavanones, naringenin (NAR), and hesperetin (HES) are bioactives with proven antioxidant and anti-inflammatory properties. Nevertheless, there are still no data about flavanone’s influence and its potential effects on the healthy aging process and improving pituitary functioning. Thus, using qPCR, immunoblot, histological techniques, and biochemical assays, our study aimed to elucidate how citrus flavanones (15 mg/kg b.m. *per os*) affect antioxidant defense, inflammation, and stress hormone output in the old rat model. Our results showed that HES restores the redox environment in the pituitary by down-regulating the nuclear factor erythroid 2-related factor 2 (Nrf2) protein while increasing kelch-like ECH-associated protein 1 (Keap1), thioredoxin reductase (TrxR1), and superoxide dismutase 2 (SOD2) protein expression. Immunofluorescent analysis confirmed Nrf2 and Keap1 down- and up-regulation, respectively. Supplementation with NAR increased *Keap1*, *Trxr1*, glutathione peroxidase (*Gpx*), and glutathione reductase (*Gr*) mRNA expression. Decreased oxidative stress aligned with NLRP3 decrement after both flavanones and glycogen synthase kinase-3 (GSK3) only after HES. The signal intensity of adrenocorticotropic hormone (ACTH) cells did not change, while corticosterone levels in serum decreased after both flavanones. HES showed higher potential than NAR in affecting a redox environment without increasing the inflammatory response, while a decrease in corticosterone level has a solid link to longevity. Our findings suggest that HES could improve and facilitate redox and inflammatory dysregulation in the rat’s old pituitary.

## 1. Introduction

Male aging is a progressive and gradual process characterized by low free testosterone, the main characteristic of the aging male hormonal status [1,2]. Specifically, aging of the pituitary gland is a multi-vector process that includes changes in hormonal and neuronal inputs to the gland itself, in addition to various structural and functional alterations occurring within the pituitary tissue. In the broader context, endocrine deficiency is associated with a progressive decline in pituitary function that, in turn, may contribute to senescence [3]. In parallel, degenerative processes affect pituitary-related regulatory limbic, hippocampal, and hypothalamic neurons and synapses, followed by compensatory gliosis [2,4]. During aging, lesions of the pituitary gland tissue vary and may include loss of pituitary endocrine cells, cyst development, fibrosis associated with chronic inflammation, distended, blood-filled vessels in the pars distalis, hemorrhage, or thrombosis [5]. Aged pituitary glands show an accumulation of oxidative products that further contribute to aging manifestation [6]. These suggest that a local redox imbalance may cause oxidative damage to cells of the hypothalamic–pituitary axis [3,7]. Generated reactive oxygen species (ROS) can lead to a rise in apoptosis of adenohypophysis cells, causing direct cellular damage but also impairment in protein function and overall hormone production with aging [3,8,9]. Studies with old rodents indicate their elevated circulating adrenocorticotropic hormone (ACTH) and glucocorticoid levels and increased release of corticotropin-releasing hormone from the hypothalamus [10]. The hyperplastic corticotrophs (ACTH cells) were observed to be mainly peripherally localized in the pituitary of old male rats [11]. In the present study, we remain focused on pituitary ACTH cells, considering their crucial role as a functional module of the hypothalamic–pituitary–adrenal (HPA) axis in affecting stress response/metabolism in advanced age.

In stress conditions, activation of nuclear factor erythroid 2-related factor 2 (Nrf2) is mediated by various exogenous and endogenous stressors such as electrophilic agents and ROS [12,13]. Nrf2 transits to the nucleus when activated, binding to antioxidant response elements (AREs) in the promoter region of genes encoding antioxidant enzymes (superoxide dismutase (SOD), catalase (CAT), glutathione reductase (GR), and peroxidase (GPx), thioredoxin (Trx) and its reductase (TrxR) system) and cytoprotective proteins. In addition, TrxR and Nrf2 signaling are crucial in regulating inflammation by modulating the transcription of antioxidants and suppressing inflammatory cytokines [14]. Besides its role in maintaining physiological cellular redox homeostasis, Nrf2 is considered a double-edged sword since overexpression of the Nrf2 leads to the development of various cancers, such as breast and prostate cancers [15,16]. Compounds that can control Nrf2 expression by maintaining it in the physiological range in healthy organisms, and preventing its constitutive activation in cancer, are of great importance [17]. Inflammation is intrinsically linked to oxidative stress, as ROS can directly or indirectly activate transcription factors such as nuclear factor kappa-light-chain-enhancer of activated B cells (NF-κB), which can promote inflammation and inflammaging [3,18]. Glycogen synthase kinase-3 (GSK3) has been proven to be a pivotal player within oxidative stress and inflammation networks, inhibiting the antioxidant function of Nrf2 and encouraging the inflammatory role of NF-κB [19]. Indeed, the active form of GSK3 is responsible for suppressing the expression of transcription factors required to overcome oxidative stress and inflammation [20]. GSK3 is a potent regulator of inflammation, while GSK3 inhibition protects from inflammatory conditions in animal models [21]. At the same time, the large varieties of stimuli fueling inflammaging converge on the activation of NF-κB and NLR family pyrin domain containing 3 (NLRP3) inflammasome, responsible for the production of inflammatory molecules. Together, Nrf2-Keap1, GSK3, NF-κB, and NLRP3 constitute a crucial regulatory mechanism in neuroinflammation and inflammaging [18,19].

In our previous papers, we showed that citrus flavanones naringenin (NAR), hesperetin (HES), and lemon extract have great potential in the restoration of the redox environment and alleviation of oxidative stress in old-aged rats [22,23,24]. Namely, by modulating the redox environment, these polyphenols up-regulated vitagene Sirtuin 1 (Sirt1) expression in thyrotropic cells [25,26,27], and improved thyroid gland sensitivity to thyroid-stimulating hormone (TSH) and overall liver health. Interestingly, studies regarding oxidative stress in the pituitary gland are scarce and mainly focused on the effects of alcohol, heavy metals, or moderate exercise [28,29,30]. However, our results suggest that soy isoflavone genistein significantly affected the HPA axis and decreased circulating ACTH and corticosterone in andropausal rats [31]. When it comes to the effects of citrus flavanones in the old pituitary, especially their relation to ACTH cell function, oxidative stress, and inflammation, there are no data so far.

Preliminarily speaking, the significance of the results presented herein regarding HES application in old-aged rats lies in the physiological improvement of the pituitary redox environment reflected in kelch-like ECH-associated protein 1 (Keap1) up-regulation, which consequently quenches excessive Nrf2 and directs its proteasomal degradation. Even the oxidative stress and inflammation are intertwined and contextualized as the Nrf2-Keap1/GSK3/NF-κB/NLRP3 regulatory loop (mechanism); these are the first data about this phenomenon in the old pituitary gland, through which we shed light on a notable potential of the plant-based, economical, and easily accessible nutraceuticals usage in the context of the pituitary health with advanced age.

## 2. Results

### 2.1. Treatment with NAR and HES Affects Nrf2 Immunofluorescent Signal, Protein, and Gene Expression in the Old Pituitary

**Figure 1:**
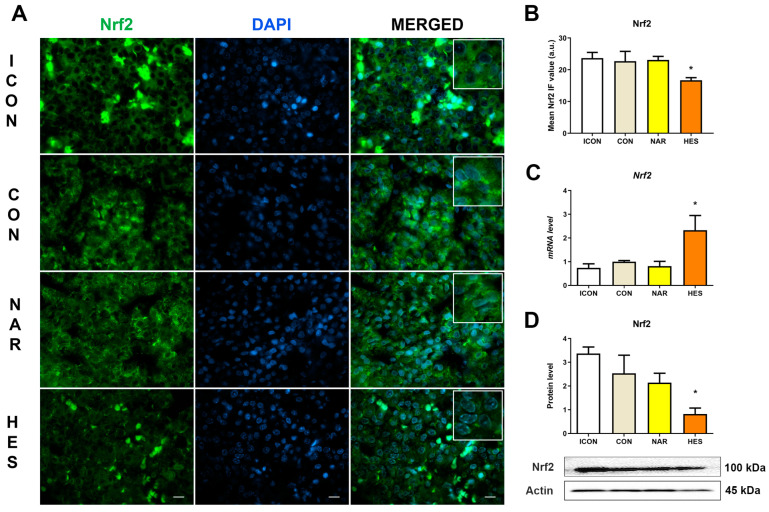
The effects of citrus flavanones on Nrf2 in the pituitary of old rats. Immunofluorescent staining of Nrf2, green and DAPI, blue signal (**A**), the mean Nrf2 IF value (**B**), the relative level of Nrf2 mRNA (**C**), and Nrf2 protein expression (**D**). Insets in the white boxes represent magnified parts of the same image, showing Nrf2 localization. The intense fluorescence spots on the micrographs represent erythrocytes and do not represent Nrf2. 63× magnification, bar = 20 μm. Each value represents mean ± SD, *n* = 4; statistics: one-way ANOVA, Dunett’s multiple comparison *post hoc* test, * *p* < 0.05 citrus flavanone vs. CON rats. ICON, intact control; CON, control sunflower oil; NAR, naringenin; HES, hesperetin.

Nrf2 is a master regulator of antioxidant and cytoprotective pathways. Its activation or inhibition leads to a series of alterations in the gene expression of molecules necessary to cope with oxidative stress. What we observed was that HES treatment induced an increase in Nrf2 gene expression by 132% and, at the same time, decreased its protein expression by 68% (Figure 1C,D). To confirm this, we applied the IF analysis of Nrf2 (counterstained with DAPI) to see the exact localization of Nrf2. Our analysis showed that the Nrf2 IF signal in ICON-, CON-, and NAR-treated groups was located in the cytoplasm of pituitary cells (Figure 1A). At the same time, we noticed a reduced signal in HES-treated pituitary sections (Figure 1A). Our IF analysis aligns with Nrf2 protein expression, which supports the thesis of a more reduced environment in the pituitary gland.

### 2.2. Treatment with NAR and HES Affects Keap1 Immunofluorescent Signal, Protein, and Gene Expression in the Old Pituitary

**Figure 2:**
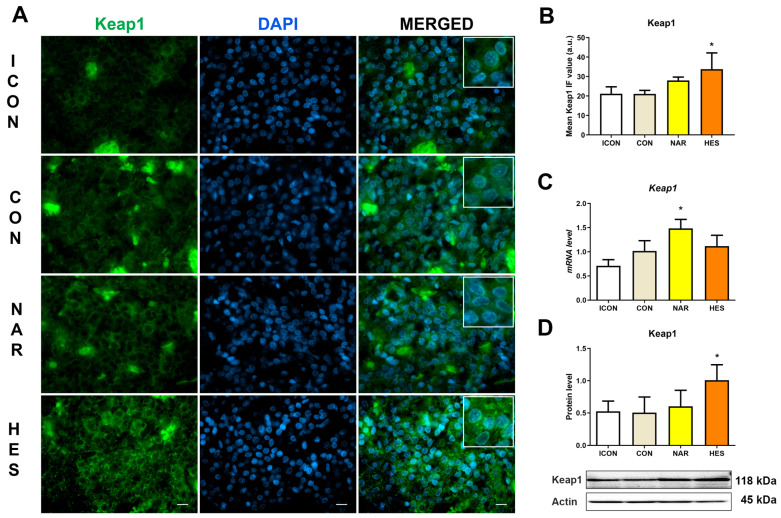
The effects of citrus flavanones on Keap1 in the pituitary of old rats. Immunofluorescent staining of Keap1, green and DAPI, blue signal (**A**), the mean Keap1 IF value (**B**), the relative level of Keap1 mRNA (**C**), and Keap1 protein expression (**D**). Insets in the white boxes represent magnified parts of the same image, showing Keap1 localization. The intense fluorescence spots on the micrographs represent erythrocytes and do not represent Keap1. 63× magnification, bar = 20 μm. Each value represents mean ± SD, *n* = 4; statistics: one-way ANOVA, Dunett’s multiple comparison *post hoc* test, * *p* < 0.05 citrus flavanone vs. CON rats. ICON, intact control; CON, control sunflower oil; NAR, naringenin; HES, hesperetin.

To assess the functional status of the Nrf2 protein, we tested the gene and protein expression of its negative regulator, Keap1. In physiological conditions, Keap1 is attached to Nrf2, which targets Nrf2 for proteasomal degradation. At the same time, in the stage of oxidative stress, Nrf2 detaches from Keap1, translocates to the nucleus, and activates the antioxidant enzyme expressions. Our results showed that NAR increased Keap1 gene expression by 46%, while HES treatment increased Keap1 protein expression by 200% (Figure 2C,D). Keap1 IF analysis showed that an intensive Keap1 IF signal was located in the cytoplasm of most cells in the pituitaries after treatment with HES (Figure 2A). In contrast, Keap1 IF signals in ICON, CON, and NAR were moderate and found in several cells’ cytoplasm (Figure 2A). A significant increase in Keap1 led to a physiological decrease in Nrf2 protein expression, suggesting a less oxidative environment.

### 2.3. Treatment with NAR and HES Affects Antioxidant Enzyme Gene and Protein Expressions in the Old Pituitary

**Figure 3:**
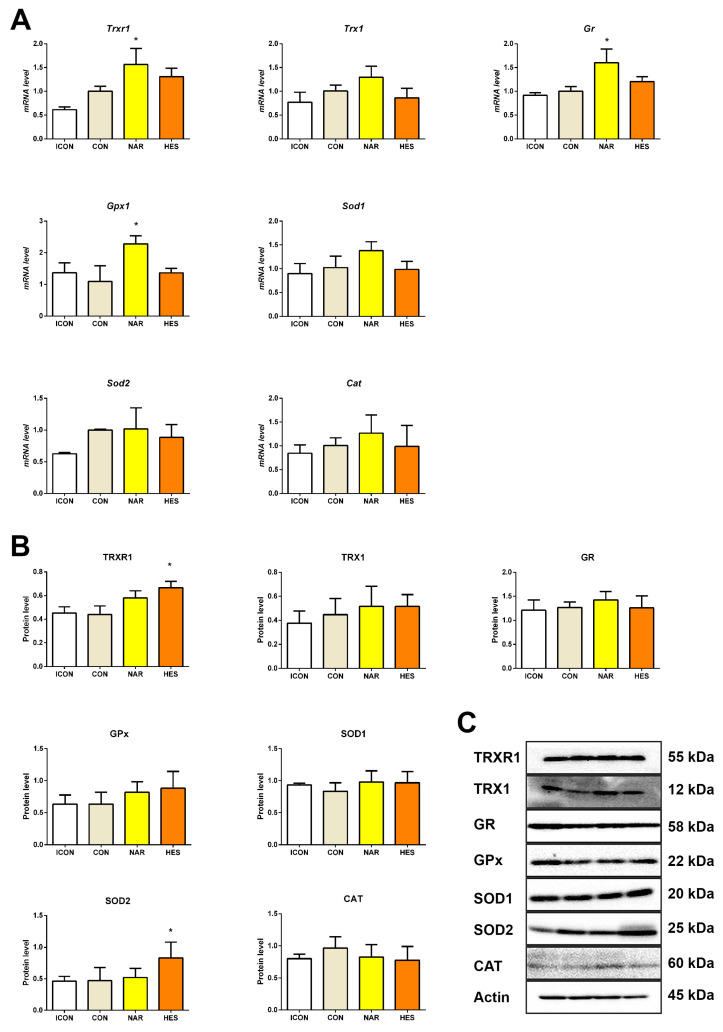
The effects of citrus flavanones on antioxidant enzymes expressions in the pituitary of old rats. Thioredoxin reductase 1 (*Trxr1*), thioredoxin 1 (*Trx1*), glutathione reductase (*Gr*), glutathione peroxidase 1 (*Gpx1*), superoxide dismutase 1 (*Sod1*), superoxide dismutase 2 (*Sod2*), and catalase (*Cat*) gene expression (**A**), TRXR1, TRX1, GR, GPx, SOD1, SOD2, and CAT protein expression (**B**), and protein profile (**C**). Each value represents mean ± SD, *n* = 4; statistics: one way ANOVA, Dunett’s multiple comparison post hoc test, * *p* < 0.05 citrus flavanone vs. CON rats. ICON, intact control; CON, control sunflower oil; NAR, naringenin; HES, hesperetin.

Gene expression analysis of the antioxidant enzymes can give us information on the mRNA level of the enzymes involved in antioxidant defense. After treatment with NAR, *Trxr1*, *Gpx*, and *Gr* gene expression increased by 55, 109, and 59%, respectively (Figure 3A), while all other targets did not alter (Figure 3A). Gene expression of examined antioxidant enzymes remained unchanged after HES treatment (Figure 3A).

Immunoblot analysis is informative regarding the protein abundance of the main actors in the antioxidant defense system. Namely, only after the HES treatment, protein levels of TrxR1 and SOD2 were up-regulated by 51% and 76%, respectively (Figure 3B). All other examined parameters did not change, likewise after NAR (Figure 3B).

### 2.4. Treatment with NAR and HES Affects Collagen Abundance, While Inflammatory Markers Remain Unchanged in the Old Pituitary

**Figure 4:**
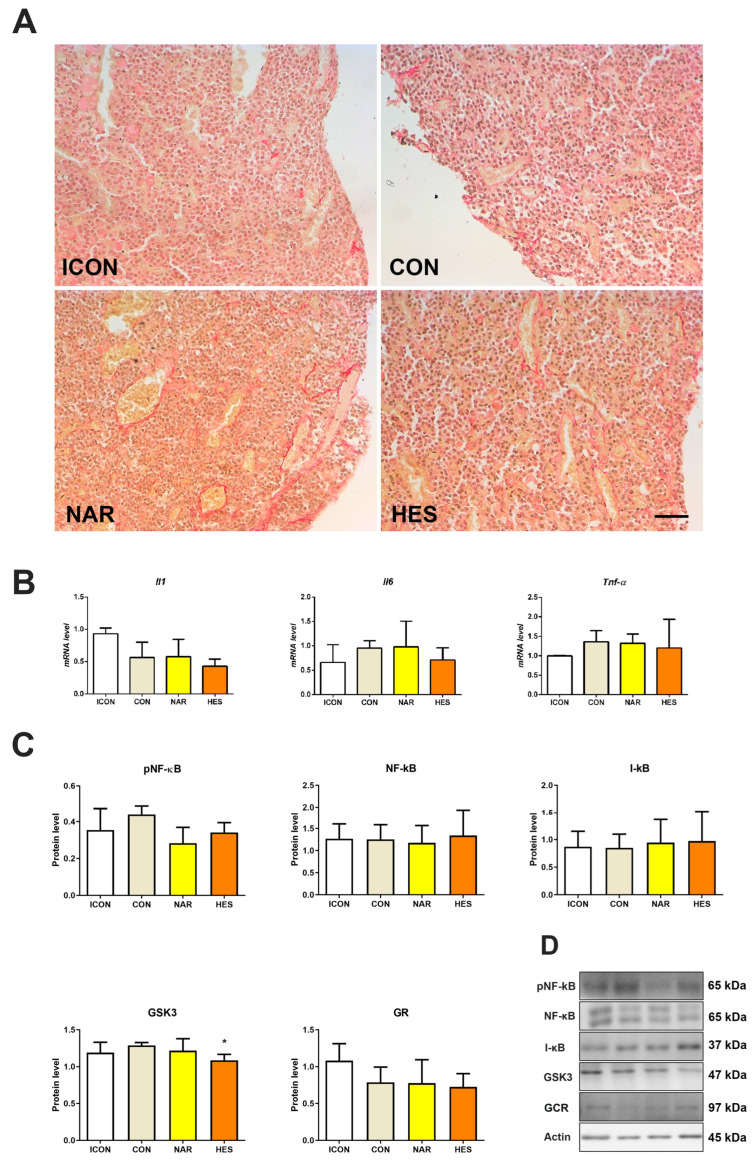
The effects of citrus flavanones on collagen accumulation and inflammatory pathway in the pituitary of old rats. Picro-Sirius Red staining of collagen (**A**), the relative level of *Il1*, *Il6,* and *Tnf-α* mRNA (**B**), protein expression of pNF-κB, NF-κB, I-κB, GSK3, and GCR (**C**), and protein profile (**D**). 20× magnification, bar = 50 μm. Each value represents mean ± SD, *n* = 4; statistics: one-way ANOVA, Dunett’s multiple comparison post hoc test, * *p* < 0.05 citrus flavanone vs. CON rats. ICON, intact control; CON, control sunflower oil; NAR, naringenin; HES, hesperetin.

Picro-Sirius Red staining is used to visualize collagen accumulation and fibrosis onset in tissue sections (Figure 4A). In both the ICON and CON control groups of aged rats, some small amount of collagen is distributed in the periphery of the pituitary pars distalis, mainly between the cell clusters and around the blood vessels (Figure 4A). Upon the treatment with citrus flavanones (NAR and HES), collagen accumulation is observed around the dilated blood vessels and between the cell clusters (Figure 4A). Of all examined parameters related to inflammation, neither NAR nor HES affected their gene expression (Figure 4B). Protein expression of GSK3 decreased by 16% (Figure 4C), while protein expression of other examined parameters did not change after the treatment with citrus flavanones (Figure 4C).

### 2.5. Treatment with NAR and HES Affects NLRP3 Expression in the Old Pituitary

**Figure 5:**
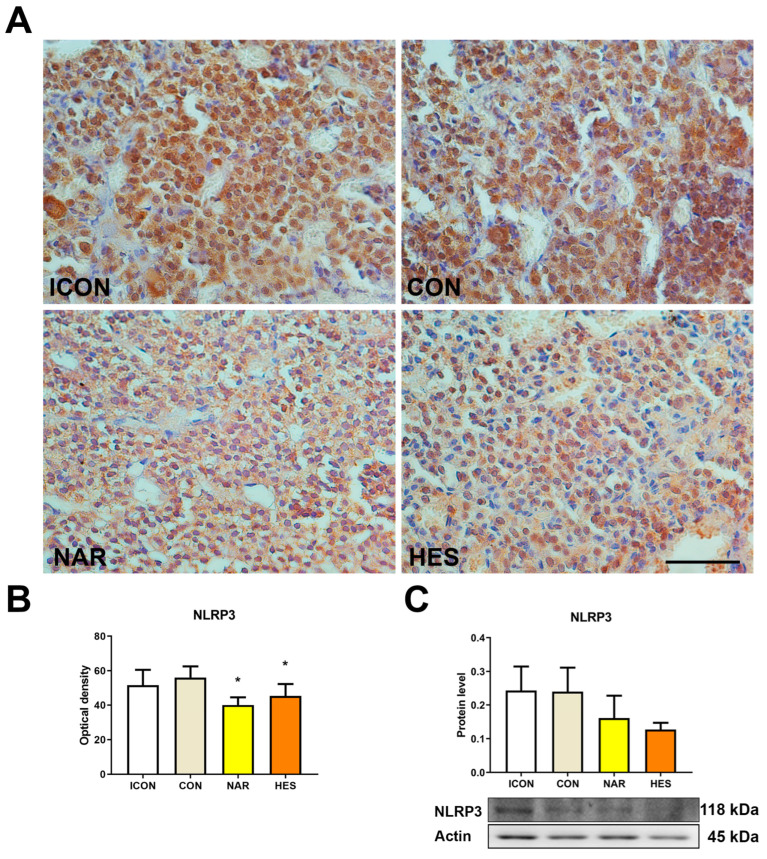
The effects of citrus flavanones on NLRP3 expression in the pituitary of old rats. Immunohistochemical staining of NLRP3 (**A**), optical density of NLRP3 (**B**), and protein expression of NLRP3 (**C**). 40× magnification, bar = 25 μm. Each value represents mean ± SD, *n* = 4; statistics: one-way ANOVA, Dunett’s multiple comparison post hoc test, * *p* < 0.05 citrus flavanone vs. CON rats. ICON, intact control; CON, control sunflower oil; NAR, naringenin; HES, hesperetin.

The NLRP3 inflammasome is a multiprotein complex that plays a pivotal role in regulating the innate immune system and inflammatory signaling, mediating the secretion of proinflammatory cytokines in response to cellular damage. Our results showed that NAR and HES decreased the immunohistochemical optical density of NLRP3 by 28 and 20%, respectively (Figure 5A,B). Even the immunoblot analysis did not show statistically significant values; the NLRP3 protein profile followed the same lowering trend after both flavanones (Figure 5C).

### 2.6. Treatment with NAR and HES Do Not Affect ACTH Signal Intensity in the Old Pituitary

**Figure 6:**
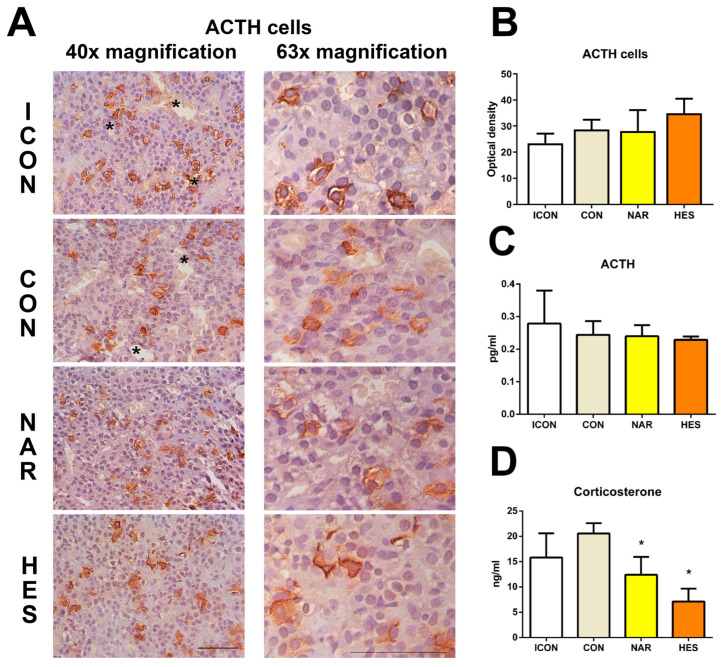
The effects of citrus flavanones on corticotroph cells in the pituitary, ACTH, and corticosterone concentration of old rats. Immunohistochemical staining of ACTH (**A**), optical density of ACTH cells (**B**), plasma level of ACTH (**C**), and corticosterone level in serum (**D**). Asterisk (*) highlights dilated blood vessels with ACTH cells around them. 40× and 63× magnification, bar = 25 and 20 μm. Each value represents mean ± SD, *n* = 4; statistics: one-way ANOVA, Dunett’s multiple comparison post hoc test, * *p* < 0.05 citrus flavanone vs. CON rats. ICON, intact control; CON, control sunflower oil; NAR, naringenin; HES, hesperetin.

In control-aged rats (ICON and CON), ACTH-immunopositive cells were mainly distributed in the periphery of the pituitary *pars distalis*, constituting small groups between or close to dilated blood vessels (asterisk, Figure 6A). The ACTH cells were morphologically polygonal or prolate in shape, occasionally with elongated cytoplasmic protrusions penetrating between neighboring cells (Figure 6A). In a naringenin-treated group of old-aged rats, ACTH cells were uniformly distributed throughout the *pars distalis* and resembled the shape of the ACTH cells in control groups (Figure 6A). Upon HES treatment of aged rats, ACTH cells’ uniform distribution and characteristic shape were maintained; however, their IHC signal, reflecting the hormonal content in the cells, looked lower (Figure 6A). This may suggest that some cells produce more ACTH after the HES-treatment group (Figure 6A,B). Nevertheless, our quantitative analysis showed no difference in the signal intensity of ACTH cells in treated groups compared to control values (Figure 6B).

### 2.7. Treatment with NAR and HES Affects the Serum Level of Corticosterone but No ACTH Level in Old Rats

Levels of plasma ACTH and serum corticosterone were measured biochemically, and they are essential parameters for testing the pituitary–adrenal axis function (Figure 6C). The level of ACTH in all examined groups was almost the same. The level of corticosterone in the ICON group was 15.81 ng/mL, while in the CON group, the value was 20.58 ng/mL (Figure 6D). After treatment with NAR or HES, the serum level of corticosterone was 12.41 ng/mL and 7.00 ng/mL, respectively (Figure 6D). So, the corticosterone level after NAR decreased by 40%, while after HES, it was 66% lower than the CON values (Figure 6D).

## 3. Discussion

Our study has revealed that HES, but not NAR, can improve a reduced redox milieu in the old pituitary. HES showed the most substantial effect on TrxR1 and Keap1 up-regulation, followed by the down-regulation of Nrf2, GSK3, and NLRP3 protein expression. At the same time, NAR affects the gene expression of *Keap1*, *Trxr1*, *Gpx*, and *Gr* enzymes involved in the antioxidant defense and decreases expression of NLRP3 protein in the pituitary glands of old rats. Both citrus flavanones decreased serum corticosterone concentration without affecting the pituitary’s ACTH level or inflammation signaling. Accumulation of the collagen around blood vessels was observed after both flavanones. Our results suggest that HES (and NAR), by attuning oxidative stress, could help the pituitary gland, without interruption, maintain its master endocrine regulatory function in old age.

Considering the worldwide consumption of citruses and the lack of available data concerning the effects of NAR and HES on pituitary function, we aimed to evaluate their outcomes in the old rats. We combined molecular biology results with structural data obtained with immuno-histochemical/-fluorescent staining regarding redox parameters, inflammatory signaling, and hormonal levels. The rationale for this study is evidence of the existence of the oxidative and proinflammatory Nrf2-Keap1/GSK3/NF-κB/NLRP3 regulatory loop [19], which is unexplored so far in the model of natural old age.

HES exerted one of the most significant results in the study by increasing Keap1 and decreasing Nrf2 protein expression. This effect is noteworthy, considering Keap1 constantly targets Nrf2 for proteasomal degradation under normal, reduced conditions [32]. Keap1 also acts as a sensor for Nrf2-activating compounds, which target the key Cys151 residues in Keap1, causing the protein to undergo conformational changes [33]. Despite HES’s high binding affinity to Keap1 [23], it does not possess prooxidant properties that could activate it, leading to its detachment from Nrf2. This result aligns with unchanged antioxidant enzyme gene expression regulated by Nrf2, observed after HES treatment. Thus, we support the thesis of a more reduced environment in the old pituitary as a positive effect.

The pituitary gland is a master endocrine gland, which develops during week four of fetal development in humans and from the 12th gestational day in rats [34,35]. Such a substantial organ has to have robust redox-sensitive regulation and antioxidant protection like the TrxR1/Trx1 system. An increase in TrxR1 protein expression after HES treatment leads to a higher capacity for Trx1-mediated disulfide reduction, de-nitrosylation, and aldehyde–protein adduct formation [3,33]. This increment helps to suppress alterations in peptide hormone synthetic machinery by decreasing ROS-mediated cellular damage. Trx enzymes recognize the oxidized form of their target proteins with higher selectivity than their corresponding reduced forms [36]. After HES treatment, higher TrxR1 expression keeps Cys151 in Keap1 in reduced form (attached to Nrf2) [33]. Thus, an improved TrxR1/Trx1 system can improve overall pituitary cell health and hormone production with aging [3].

In addition, TrxR1 is an essential negative regulator of Nrf2, meaning that loss of TrxR1 activity leads to Nrf2 activation and vice versa [33]. Interestingly, several polyphenolic compounds have dual but opposite effects on these two proteins. Compounds such as curcumin, quercetin, ellagic acid, isothiocyanates, and sulforaphane possess both inhibitory activities against TrxR1 and, at the same time, the ability to activate Nrf2 by covalently modifying reactive Cys residues in Keap1 in vitro [33]. Nonetheless, our in vivo study suggests this is a less possible scenario due to the up-regulation of Keap1 and TrxR1. In vivo studies differ from in vitro studies due to complex interaction and metabolic transformations of the examined compounds.

Up-regulation in the mitochondrial enzyme SOD2 is another positive outcome after HES treatment. Even though we showed a decrease in Nrf2 and unaltered NF-κB protein expression after HES, a possible explanation could involve increased Sirt1 expression in the same organ and model we showed in our previous study [23,37]. Namely, Sirt1, by deacetylation, enhances SOD2 expression in mitochondria by activating forkhead box protein O (FOXOs), resulting in increased removal of O_2_^−^ or attenuating mitochondrial ROS production [38,39]. Additionally, resveratrol and quercetin have been found to enhance SOD2 expression mediated by Sirt1, thus mitigating oxidative stress in different animal models [40]. SOD2, by eliminating O_2_^−^ generates O_2_ and H_2_O_2_, which GPx and CAT scavenge. Both enzymes are crucial in the host’s defense against oxidative stress and cell protection against H_2_O_2_ toxicity [41]. In our study, GPx and CAT expression did not change after treatment with NAR and HES, proposing no significant H_2_O_2_ insult in the old pituitary.

Furthermore, our study revealed a decrease in GSK3 protein expression after HES. This result is beneficial since the active form of GSK3 promotes the production of proinflammatory cytokines such as IL-1β, TNF-α, and IL-6 [20,21], whose gene expressions were unchanged after citrus flavanones. In addition, GSK3 down-regulation reduces its proinflammatory function since GSK3 directly stimulates NF-κB signaling [21,42]. Besides inflammation, GSK3 maintains redox equilibrium in the body. It does so by stimulating the production of ROS in the mitochondria and increasing the inner mitochondrial membrane permeability to detrimental molecules [43]. Activated GSK3 down-regulates the expression of Nrf2 and its ability to bind to ARE in cerebral ischemia and reperfusion injury [44]. Overall, down-regulating GSK3 and up-regulating TrxR1 and SOD2 after HES in our study suggest that HES can protect cells from ROS and inflammation, increasing pituitary cell survival.

After treatment with citrus flavanones, we did not observe fibrosis in the aged human adenohypophyses [45]. However, we observed the collagen positivity surrounding the blood vessels, prominently seen after NAR or HES, without affecting the pituitary parenchyma. Fibrosis is a repair mechanism that becomes activated after the body is stimulated by inflammation or physical damage. Activating the NF-κB signaling pathway and increasing IL-6 is probably the critical mechanism in developing pituitary fibrosis and inflammaging process in older adults [46,47]. NAR and HES did not alter inflammatory signaling mediated by NF-κB, or the pNF-κB/NF-κB ratio; thus, this change is probably a result of better blood supply and organ perfusion after our treatment. In general, the anti-inflammatory effect of GCR is attributed to the suppression of inflammatory genes and NFkB [48,49]. However, its protein content remained unchanged under HES and NAR treatment, confirming our assumption that inflammation was not affected and that the observed morphological changes are rather the result of increased blood supply to the pituitary gland. It is important to emphasize that polyphenols can pass the blood–brain barrier. Namely, metabolites of polyphenols are hydrosoluble molecules, unlike their aglycone form [50,51], meaning that after their complete metabolism in the liver, they can be transported all over the organism by blood. Although pituitary capillaries are part of the central nervous system vascularization, their blood vessel components have specific properties that differ from vascular networks in other brain areas due to fenestrated capillaries, allowing the passing of hormones, nutrients, etc. [52]. Polyphenol metabolites can reach pituitary cells, where they exert protective effects by modulating oxidative stress and inflammation [53].

A decrement in NLRP3 expression in the pituitary is in line with the previous results. Inflammasomes have been identified as essential drivers of sterile inflammation, and there is accumulating evidence that NLRP3 activation might also play an important role in the aging process [54]. It has been revealed that the cytosolic ROS induced by NADPH is responsible for the activation of the NLRP3 inflammasome [55], while in our study, lower NLRP3 expression is in line with a better redox environment in the pituitary. Our result is a positive outcome since microglial NLRP3 inflammasome activation up-regulates the pituitary glands’ inflammatory cytokines IL1/IL18, and induces prolactinomas [56] or other impairments that could compromise normal pituitary functioning. HES and NAR showed positive neuroprotective effects by down-regulating the NLRP3 inflammasome activation in the brain [57,58]. However, the applied doses were 3–6 times higher than ours. Therefore, this nominates citrus flavanones as nutritionally achievable supplements that can reduce pituitary inflammaging and age-related pathologies at the population level.

The main effect after HES treatment is the activation of two of the three vitagenes essential for the neuronal function and longevity phenomenon [25,59,60,61], TRXR1, and Sirt1 [23]. This result promotes HES as a desirable supplement in alleviating ROS-induced diseases and improving aging-related malfunction of pituitary-related diseases. NAR also affected examined parameters since it increased *Keap1*, *Trxr1*, *Gpx*, and *Gr* mRNA expression, while there is an increasing trend regarding TrxR1, Trx1, GR, GPx, and Keap1 protein levels. Citrus flavanones in our study exerted different effects since HES, compared to NAR, possesses one hydroxyl less and one methyl group more, making it less prooxidant than NAR. This suggests that even though it has a similar structure, NAR, in comparison with HES, exerts different biological effects, but mainly in the same direction of change. However, low polyphenol doses in our study follow the hormesis concept, meaning preconditioning with HES or NAR can help the old pituitary handle upcoming stress better.

As we described, citrus flavanones (HES more than NAR) protect pituitary gland synthetic/secretory and overall regulatory pathways from ROS-mediated damage of peptide/protein components. Namely, ACTH cell signal intensity in NAR- and HES-treated old rats did not change, but a certain accumulation of hormonal content in these cells upon HES application suggests their synthetic activation. This result may be related to a significantly reduced corticosterone (even more than compared to the NAR group) and the initiation of a feedback mechanism. Generally, ACTH release is mediated by the adenylate cyclase protein kinase (PKA) system [62], and oxidants, if present for a longer time or in higher concentrations, can inhibit PKA phosphorylation by directly oxidizing a reactive cysteine in its catalytic subunit [63]. However, in our study, the serum ACTH and corticotroph IHC signals remained unchanged, suggesting some potential issues in the central regulation at the pituitary level. Namely, it was shown that with aging in rats and humans, there is an impairment in the ACTH cell’s response to corticosterone, leading to decreased HPA axis sensitivity to the corticosterone/cortisol feedback loop, which is all accompanied by degenerative processes in the hippocampal and hypothalamic neurons [2,4,64,65]. Therefore, even with lower corticosterone, ACTH remained unaltered in serum and corticotrophs. Additionally, a decrease in corticosterone is in line with the effects of soy isoflavones in the animal model of andropause [66,67]. What deserves attention in future studies that will follow up on this one is examining the effects of citrus flavanones on the activity of adrenocortical steroidogenesis enzymes. Indeed, we did not analyze the potential impact of citrus flavanones on adrenal glands since that was not the main focus of our study. However, by disrupting corticosteroidogenesis, potentially due to inhibiting of hormone-generating machinery, citrus flavanones could modulate and alter stress hormone levels in old rats. Further, we advocate decreasing stress hormones after NAR and HES as a positive outcome since increased cortisol level is associated with metabolic, somatic, and psychiatric conditions related to aging [68,69], while proinflammatory cytokines secreted in many of these conditions may act on the HPA axis, increasing glucocorticoid secretion [69]. Studies showed that lower cortisol in humans and corticosterone in rats positively correlate with mammalian longevity [70,71,72] due to the ability of these hormones to reduce the cellular production of free radicals, which is observed in long-lived species [72]. Revived thyrotropic activity, which has already been described [23] and showed positive effects on corticosterone, indicates citrus flavanones are potential longevity-modulating molecules.

In general, the results obtained in our study are valuable since we analyzed two processes that can have detrimental effects on the pituitary gland: oxidative stress and inflammation. After HES and NAR, inflammation stayed at a steady state level, and the redox environment even improved. A decline in endocrine function characterizes the old pituitary; still, it remains an essential player in maintaining the organism’s homeostasis. However, we showed that our treatments do not influence ACTH cell morphology, IHC signal, and ACTH blood level, even with the provided positive environment. NLRP3 expression was down-regulated, which directly reduced the generation of the local proinflammatory cytokines, which could contribute to HPA axis regulation in a paracrine manner. This is positive since prolonged inflammatory response in aged animals might be linked to dysregulated pituitary cytokine interactions, leading to a rise in serum corticosterone levels [73]. Another benefit of our study is that it opens another new research focus, directed towards the adrenal gland, considering the decline in corticosterone serum levels. In the end, with the other positive outcomes of the present and our previous works [22,23,24], we are persistently getting closer to elucidating the effects of citrus flavanones on extending the life span.

## 4. Materials and Methods

### 4.1. Experimental Animals

Two-year-old male Wistar rats used in the experiment were bred and housed in the Unit for Experimental Animals at the Institute for Biological Research “Siniša Stanković”—National Institute of the Republic of Serbia, Belgrade, Serbia, in cycles of 12 h light and 12 h dark and constant temperature (21 ± 2 °C) conditions, and had access to a chow diet and water *ad libitum*. All animal procedures complied with Directive 2010/63/EU on protecting animals used for experimental and other scientific purposes and were approved by the Ethical Committee for the Use of Laboratory Animals of IBISS, University of Belgrade (No 2-12/12).

### 4.2. Dosage Regimen

At the beginning of the experiment, we randomly divided the old-aged rats into four experimental groups (*n* = 6 per group). Treated groups of animals received *per os* 15 mg/kg b.m. of citrus flavanones naringenin (NAR) or hesperetin (HES) mixed with sunflower oil. To conduct a nutritionally relevant study, we avoided gavage, and the mixture was applied to the oral cavity, considering the role of oral microbiota in consumed flavanone biotransformation. The applied volume of the mixture was 300 μL *per* animal by syringe directly to the oral cavity. The control group (CON) received the same volume of the vehicle, while physiologically intact controls (ICON) represent physiologically intact animals. The treatments were administered daily for four weeks.

### 4.3. RNA Isolation, cDNA Transcription and Real-Time PCR

Total pituitary RNA was isolated using TRIzol (Invitrogen, Carlsbad, CA, USA) and purified using the RNeasy mini-kit (74106, QIAGEN, Hilden, Germany) following the manufacturer’s instructions. The cDNA was synthesized with a High-Capacity cDNA Reverse Transcription Kit (4368813, Applied Biosystems, Vilnius, Lithuania) with 500 ng of RNA. PCR amplification of cDNAs was performed in a real-time PCR machine ABI Prism 7000 (Applied Biosystems, Waltham, MA, USA) with SYBRGreen PCR master mix (4309155, Applied Biosystems, Waltham, MA, USA). The program included the following conditions: 3 min at 95 °C, followed by 40 cycles of 15 s at 95 °C, 30 s at 60 °C, and 30 s at 72 °C. The list of primers used is shown in Table S1. Melting curve analysis was used to confirm gene-specific amplification. Nuclease-free water was used as a negative control instead of a cDNA template from the individual samples to test if there was no residual genomic DNA. The expression level of each gene was calculated using the formula 2 − (ΔΔ^Ctexp^ − ΔΔ^Ctcontrol^), where ΔCt is different between the cycle threshold value of the gene of interest and the cycle threshold value of *Gapdh* or *Hprt* as a reference gene. All of the data were calculated from duplicate reactions. RNA data are presented as average relative levels vs. *Gapdh* ± SD for antioxidant enzymes or *Hprt* ± SD for inflammatory markers.

### 4.4. Protein Isolation for Western Blot

Total protein isolation from the rat pituitary was performed using TRIzol following the manufacturer’s protocol with slight modifications published by [23]. Briefly, the manufacturer’s protocol for protein isolation was followed until the precipitation of proteins from the phenol-ethanol supernatant. At this step, the protein pellet was completely dissolved in 7 M GndCl solution, and then proteins were precipitated again by adding 100% ethanol. This step was repeated, followed by a final wash of the protein pellet with 100% ethanol. After 10 min of air drying, the protein pellet was solubilized in the buffer containing 8 M Urea, 40 mM Tris pH 8, 2% SDS, and 1× Protease G inhibitor cocktail (39101.03, Serva, Heidelberg, Germany). Protein concentration in samples was measured by DC Protein Assay (#5000112, BioRad, Goettingen, Germany), using bovine serum albumin as standard.

### 4.5. SDS Polyacrylamide Gel Electrophoresis and Western Blot

Proteins were solubilized in 4× Laemmli sample buffer supplemented with 10% β-mercaptoethanol. A quantity of 15 μg of protein *per* lane was subjected to 10% or 12% SDS-polyacrylamide gel electrophoresis and transferred electrophoretically to polyvinylidene difluoride (PVDF) or nitrocellulose membranes with a semidry or wet blotting system (Fastblot B43; Bio-Rad, Goettingen, Germany). The next step was blocking of unbound sites on the membranes with 5% BSA (prior incubation with SOD1, SOD2, CAT, GPx, GR, Keap1, Nrf2, Trx1, TrxR1, pNF-kB, NF-kB, I-kB, GCR, GSK3, NLRP3, and β-Actin; Table S2) for one hour. The membranes were incubated with primary antibodies overnight at 4 °C. The list of used antibodies is shown in Table S2. After washing, blots were incubated with secondary antibodies for one hour at room temperature. Antibody binding was detected using a chemiluminescence detection system (ECL; BioRad, Goettingen, Germany). Signals were quantified by densitometry using ImageJ Image Analysis Software (v1.48).

### 4.6. Histological Sample Preparation

Pituitaries were excised and fixed in 10% formalin for 48 h, washed in tap water, then dehydrated in a series of increasing concentrations of ethanol (30–100%), enlightened in xylol, and embedded in Histowax^®^ (Histolab Product AB, Göteborg, Sweden). Sections with a thickness of 5 µm were prepared using a rotational microtome (RM 2125RT Leica Microsystems, Wetzlar, Germany).

### 4.7. Sirius Red Histochemical Staining

Sirius Red staining, used in histological analysis, is often a method of choice when there is a need to distinguish different cells from collagen. In the initial steps, the procedure involved deparaffinization (xylol) and rehydration (100–70% ethanol, distilled water) of the pituitary sections, followed by incubation in Weigert’s hematoxylin (8 min). After washing in running tap water, the sections were incubated in a Picro-Sirius Red solution (0.5 g of Sirius Red (Direct Red 80, 365548; Sigma Aldrich, Co., St. Louis, MO, USA) + 500 mL of saturated aqueous solution of picric acid) for 1 h. The next step included double washing pituitary sections in acidified water (5 mL of glacial acetic acid in 1 L of distilled water), followed by vigorous shaking to physically remove most of the water from the slides. Finally, the sections were dehydrated in three changes of 100% ethanol, cleared in xylol, and mounted in DPX (Sigma-Aldrich, Co., St. Louis, MO, USA).

### 4.8. Immunohistochemical Staining of ACTH and NLRP3

The representative sections were stained with immunohistochemical (IHC) methods according to previously described procedures [31]. After tissue deparaffinization, endogenous peroxidase activity was blocked by sections incubated with 0.3% hydrogen peroxide in methanol for 15 min. Only slides used in NLPR3 IHC analysis were exposed to heat-induced antigen retrieval by placing them in a container with citrate buffer (pH 6.0) and then heated at 750 W in a microwave oven for 10 min. Non-specific background staining was reduced by incubation with normal swine serum (X0901, Dakopatts, Glostrup, Denmark) diluted 1:10 for 45 min. For functional IHC analysis, the rabbit antisera directed against ACTH and NLRP3 (Table S2) were applied overnight at room temperature. The primary antibody was substituted with phosphate buffer saline (PBS) for the negative control of the pituitary sections. Secondary antibodies labeled with HRP were applied for one hour. After secondary antibody incubation, NLRP3 slides were incubated with the VECTASTAIN ABC Kit (PK-4001, Vector Laboratories, Inc., Burlingame, CA, USA) according to the manufacturer’s instruction. Visualization was performed using the Dako liquid diaminobenzidine tetrahydrochloride substrate chromogen system (K3468, Dako North America, Inc., Carpinteria, CA, USA) at concentrations suggested by the manufacturer. All washes and dilutions were performed using 0.1 mol/l PBS pH 7.4. Hematoxylin was used as a counterstain, and slides were mounted in DPX medium (Sigma-Aldrich, Barcelona, Spain).

### 4.9. Immunofluorescent Staining of Nrf2 and Keap1

The representative pituitary sections were stained using the immunofluorescence (IF) method, as previously described [24], with certain modifications. Namely, pituitary sections were exposed to heat-induced antigen retrieval after tissue deparaffinization and rehydration by placing them in a container with Tris-EDTA buffer (pH 9.0) for Nrf2 or citrate buffer (pH 6.0) for Keap 1, and then heated at 750 W in a microwave oven for 10 min. Then, only the samples intended for Nrf2 analysis were incubated for 15 min with PBS containing 0.2% Triton X-100 to permeabilize cells and nuclear membranes. Non-specific background staining was reduced by incubation with normal donkey serum (ab7475, Abcam, Cambridge, UK) diluted 1:10 for 45 min. For functional IF analysis, the rabbit antisera directed against the Nrf2 or Keap1 antigen was applied overnight at room temperature. A secondary antibody (Table S2) was applied for 45 min and washed 5 × 5 min with PBS 0.05% Tween20 pH 7.4. As counterstain, we used 4′,6-diamidino-2-phenylindole (DAPI; 300 nM) for 5 min and washed with PBS for 3 × 5 min. Slides were mounted in Mowiol medium (Sigma-Aldrich, St. Louis, MO, USA).

### 4.10. Image Acquisition

Digital images of the ACTH-, NLRP3-immunopositive, and Sirius Red-stained pituitary sections were taken using a LEITZ DM RB light microscope (Leica Mikroskopie & Systems GmbH, Wetzlar, Germany), a LEICA DFC320 CCD camera (Leica Microsystems Ltd., Heerbrugg, Switzerland), and the Leica DFC Twain Software (4.11.0, Leica, Wetzlar, Germany). A Zeiss Axiovert fluorescent microscope (Zeiss, Graz, Austria) was used to acquire fluorescent images of Nrf2 and Keap1 antibodies.

### 4.11. Concentration of the ACTH and Corticosterone

Blood was collected from the trunk, and separated plasma and sera samples of all the animals were stored at the same time at −70 °C until assayed. Plasma levels of ACTH were determined without dilution by the IMMULITE method (2500 ACTH, L5KAC2, DPC, Los Angeles, CA, USA), in duplicate samples within a single assay, with an intra-assay CV of 9.6%. The detection limit of the assay, defined as the concentration two standard deviations above the response at zero dose, is approximately 9.0 pg/mL. Serum corticosterone concentrations were measured without dilution by immunoassay (KGE009, R&D Systems Inc., Minneapolis, MN, USA), in duplicate samples within a single assay, with an intra-assay CV of 8.0%. The sensitivity of this corticosterone immunoassay is typically less than 27.0 pg/mL.

### 4.12. Statistical Analysis

All obtained results were analyzed using GraphPad Prism v.6 for Windows (San Diego, CA, USA). The data for the experimental groups were first tested for distribution normality with the Kolmogorov–Smirnov test. After confirmation of a Gaussian distribution and homogeneity of variance with Bartlett’s test, one-way ANOVA was used for further comparative evaluation, followed by Dunett’s *post hoc* test. The potential effect of vehicle (sunflower oil; CON) was evaluated versus untreated ICON animals. The effect of NAR or HES treatment on all examined parameters was evaluated in comparison to the values obtained for the CON group. A confidence level of *p* < 0.05 was considered statistically significant. The data are presented as means ± SD.

## 5. Conclusions

**Figure 7:**
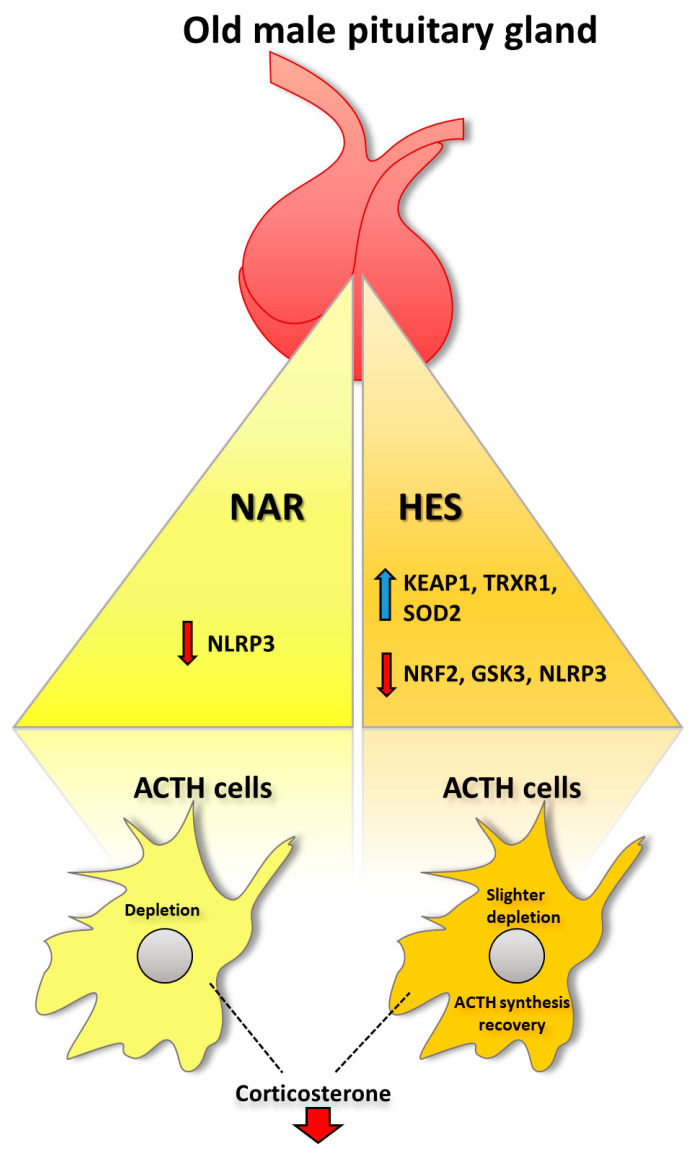
Summarized naringenin (NAR) and hesperetin (HES) effects in the pituitary gland of the old male rats. *Trxr1*, thioredoxin reductase 1; SOD2, superoxide dismutase 2; NRF2, nuclear factor erythroid 2-related factor 2; GSK3, glycogen synthase kinase-3; keap1, kelch-like ECH-associated protein 1; NLRP3, NLR family pyrin domain containing 3. Blue arrow, up-regulation; red arrow, down-regulation.

Finally, the lack of research regarding the current topic suggests that more data are needed to make a conclusive decision about how beneficial these molecules could be for pituitary function. However, with data from the recent study (summarized in Figure 7), we opened a new important question: whether citrus flavanones (and other polyphenols) can be used as nutritionally relevant molecules capable of affecting oxidative stress and inflammation in the pituitary. Considering that the pituitary gland is a master endocrine regulator, every molecule that can affect it is worth testing since it could be a new, low-cost, and already accepted food supplement for improving and facilitating pituitary redox and/or endocrine disorders in all ages.

## Data Availability

Data are contained within the article and Supplementary Materials.

## Supplementary Materials

The following supporting information can be downloaded at: https://www.mdpi.com/article/10.3390/ijms25168918/s1.
