# Supplementary material for: Citrus Flavanone Effects on the Nrf2-Keap1/GSK3/NF-κB/NLRP3 Regulation and Corticotroph-Stress Hormone Loop in the Old Pituitary
Source: Int J Mol Sci. 2024 Aug 16;25(16):8918. doi: 10.3390/ijms25168918 (PMC11354440; doi:10.3390/ijms25168918)
Supplement: Supplementary file 1 [file ijms-25-08918-s001.zip › Table S1 List of primers.pdf]

Citrus Flavanone Effects on the Nrf2-Keap1/GSK3/NF-κB/NLRP3 Regulation and Corticotroph-Stress Hormone Loop in the Old Pituitary.

Marko Miler [1*], Jasmina Živanović [1], Sanja Kovačević[2], Nevena Vidović[3], Ana Đorđević[2], Branko Filipović[1], Vladimir Ajdžanović[1]

[1]Department of Cytology, Institute for Biological Research "Siniša Stanković"- National Institute of the Republic of Serbia, University of Belgrade, Belgrade, Serbia

[2]Department of Biochemistry, Institute for Biological Research "Siniša Stanković"- National Institute of the Re-public of Serbia, University of Belgrade, Belgrade, Serbia

[3] Centre of Research Excellence in Nutrition and Metabolism, Institute for Medical Research, University of Bel-grade, Belgrade, Serbia

*Correspondence to: Marko Miler, PhD, Senior Research Associate

Institute for Biological Research "Siniša Stanković"- National Institute of the Republic of Serbia, University of Belgrade

142 Despot Stefan Blvd.

11060 Belgrade, Serbia

Phone: +381 11 2078 321

Fax: +381 11 2761 433

E-mail: marko.miler@ibiss.bg.ac.rs

Table S1. List of primer pairs used in qPCR analysis.

Gene	Primer sequence
Sod1	f: 5′-AAGCGGTGAACCAGTTGTG-3′<br>r: 5′-CCAGGTCTCCAACATGCC-3′
Sod2	f: 5′-GGTGGAGAACCCAAAGGAGA-3′<br>r: 5′-AGCAGTGGAATAAGGCCTGT-3′
Cat	f: 5′-CCAGCGACCAGATGAAGCA-3′<br>r: 5′-TGGTCAGGACATCGGGTTTC-3′
Gpx	f: 5′-TATAGAAGCCCTGCTGTCCA-3′<br>r: 5′-CAAGCCCAGATACCAGGAA-3′
Gr	f: 5′-ACGAGGAAGACGAAATGCGTGATG-3′<br>r: 5′-AGGATGAATGGCGA CGCTATTGTC-3′
Nrf2	f: 5′-GAGCGGGAGAAATCACACAGAATG-3′<br>r: 5′-CAGGAGCTGCATGCACTCATCG-3′
Txn1	f: 5′-AAGTAGACGTGGATGACTGCC-3′<br>r: 5′-CCAGAGAACTCCCCAACCTTT-3′
Txnr1	f: 5′-TCCTTTTCTTGTCCTCCCC-3′<br>r: 5′-TCTTTTCTCCTCTTCAACACAC-3′
Keap1	f: 5′-TGAACTCCTACCACCCACAC-3′<br>r: 5′-TTCCTTCTTTCCCTTGTCCC-3′
Il-1	f: 5′-AGCAGCTTTCGACAGTGAGG-3′<br>r: 5′-CTCCACGGGCAAGACATAGG-3′
Il-6	f: 5′-GTTTCTCTCCGCAAGAGACTT-3′<br>r: 5′-ATACTGGTCTGTTGTGGGTGG-3′
Tnf-α	f: 5′-GCC ACC ACG CTC TTC TGT CT-3′<br>r: 5′-CGC TTG GTG GTT TGC TAC GAC-3′
Gapdh	f: 5′-GTGGACCTCATGGCCTACAT-3′<br>r: 5′-GGATGGAATTGTGAGGGAGA-3′
Hprt	f: 5′-CAGTCCCAGCGTCGTGATTA-3′<br>r: 5′-AGCAAGTCTTTCAGTCCTGTC-3′
