# Supplementary material for: Citrus Flavanone Effects on the Nrf2-Keap1/GSK3/NF-κB/NLRP3 Regulation and Corticotroph-Stress Hormone Loop in the Old Pituitary
Source: Int J Mol Sci. 2024 Aug 16;25(16):8918. doi: 10.3390/ijms25168918 (PMC11354440; doi:10.3390/ijms25168918)
Supplement: Supplementary file 1 [file ijms-25-08918-s001.zip › Table S2 List of antibodies Rev.pdf]

Citrus Flavanone Effects on the Nrf2-Keap1/GSK3/NF-κB/NLRP3 Regulation and Corticotroph-Stress Hormone Loop in the Old Pituitary.

Marko Miler [1*], Jasmina Živanović [1], Sanja Kovačević[2], Nevena Vidović[3], Ana Đorđević[2], Branko Filipović[1], Vladimir Ajdžanović[1]

[1]Department of Cytology, Institute for Biological Research "Siniša Stanković"- National Institute of the Republic of Serbia, University of Belgrade, Belgrade, Serbia

[2]Department of Biochemistry, Institute for Biological Research "Siniša Stanković"- National Institute of the Re-public of Serbia, University of Belgrade, Belgrade, Serbia

[3] Centre of Research Excellence in Nutrition and Metabolism, Institute for Medical Research, University of Bel-grade, Belgrade, Serbia

*Correspondence to: Marko Miler, PhD, Senior Research Associate

Institute for Biological Research "Siniša Stanković"- National Institute of the Republic of Serbia, University of Belgrade

142 Despot Stefan Blvd.

11060 Belgrade, Serbia

Phone: +381 11 2078 321

Fax: +381 11 2761 433

E-mail: marko.miler@ibiss.bg.ac.rs

Table S2. List of antibodies used in Western blot (WB), immunofluorescent (IF) and immunohistochemical (IHC) analyses.

Primary antibodies used					
**Method**	**Name**	**Cat #**	**Origin**	**Dilution**	**Manufacturer**
**WB**	**SOD1**	ab13498	Rabbit	1:2000	Abcam, UK
	**SOD2**	ab13533			
	**CAT**	ab16731			
	**GPx**	ab22604			
	**GR**	ab16801			
	**Nrf2***	EP1808Y		1:1000	
	**Keap1***	ab139729			
	**Trx1**	ab133524		1:5000	
	**Trxr1**	ab124954		1:8000	
	**pNF-kB-Ser536**	93H1, #3033	Rabbit	1:1000	Cell Signaling, MA, USA
	**NF-kB**	sc8008	Mouse	1: 500	Santa Cruz, UK
	**I-kB**	sc371	Rabbit	1:250	
	**GSK3**	sc7291	Mouse	1:500	
	**GR (glucocorticoid receptor)**	PA1-511A	Rabbit	1:250	Thermo Fisher, CA, USA
	**NLRP3***	NBP2-12446	Rabbit	1:200	Novus, CO, USA
	**β-Actin**	3700S	Mouse	1:4000	Cell Signaling, MA, USA
**IF**	**Nrf2**	EP1808Y	Rabbit	1:100	Abcam, UK
	**Keap1**	ab139729			
**IHC**	**ACTH**	AFP-156102789	Rabbit	1:500	From Dr. A.F.Parlow (National Institute of Diabetes & Digestive & Kidney Diseases), USA
	**NLRP3***	NBP2-12446	Rabbit	1:50	Novus, CO, USA
**Secondary antibodies used**					
**WB**	**Anti-mouse, HRP labeled**	7076S	Donkey	1:5000	Cell Signaling, MA, USA
	**Anti-rabbit, HRP labeled**	7074S			
**IF**	**Anti-rabbit, Alexa fluor 488**	A-21206	Donkey	1:150	Thermofisher, CA, USA
**IHC**	**Anti-rabbit, HRP labeled**	P0399	Swine	1:100	DAKO, Denmark

*the same antibody used for different analysis and in different concentration
